# Synucleins and Gene Expression: Ramblers in a Crowd or Cops Regulating Traffic?

**DOI:** 10.3389/fnmol.2017.00224

**Published:** 2017-07-13

**Authors:** Alexei A. Surguchev, Andrei Surguchov

**Affiliations:** ^1^Department of Surgery, Section of Otolaryngology, Yale School of Medicine, Yale University, New Haven CT, United States; ^2^Department of Neurology, University of Kansas Medical Center, Kansas City KS, United States

**Keywords:** gene expression, synucleins, epigenetic regulation, transcription factors, translation factors, DNA methylation, histone modifications, neurodegeneration

## Abstract

Synuclein family consists of three members, α, β, and γ-synuclein. Due to their involvement in human diseases, they have been thoroughly investigated for the last 30 years. Since the first synuclein identification and description, members of this family are found in all vertebrates. Sequencing of their genes indicates high evolutionary conservation suggesting important function(s) of these proteins. They are small naturally unfolded proteins prone to aggregate, easily change their conformation, and bind to the membranes. The genes for α, β, and γ-synuclein have different chromosomal localization and a well preserved general organization composed of five coding exons of similar size. Three genes encoding synucleins are present in the majority of vertebrates, however, a variable number of synuclein genes are described in fishes of different species. An important question concerns their normal function in cells and tissues. α-Synuclein is implicated in the regulation of synaptic activity through regulation of synaptic vesicle release, while the physiological functions of two other members of the family is understood less clearly. Here we discuss recent results describing their role in the regulation of gene expression.

## Introduction

Since the discovery of the first synuclein by Maroteaux et al. (1988), the members of the synuclein family attract growing attention primarily as proteins implicated in neurodegenerative (α-synuclein) and neoplastic (γ-synuclein) diseases. The majority from almost 9,000 publications describe how these naturally unfolded proteins aggregate and propagate between neurons (α-synuclein), or cause misregulation of intracellular signaling pathways in oncological transformation (γ-synuclein). β-Synuclein, the third member of the family, received much less attention. It is less prone to form insoluble aggregates and presumably plays a protective role against α-synucleinopathies (Hashimoto et al., 2001). Since the discovery of synucleins, researchers investigate mostly α-synuclein for its association with synucleinopathies, i.e., PD, PD dementia, DLB and multiple system atrophy.

During the late 1990s the popular theory to explain mechanisms causing α-synuclein aggregation in cellular milieu was a concept of macromolecular crowding (Minton, 1993, 1998; Uversky et al., 2001). According to this concept, total cytoplasmic concentration of proteins and other macromolecules can either reach the high level of 400 g/l causing macromolecular crowding (Uversky et al., 2001, 2002; Shtilerman et al., 2002) or be subjected to excluded volume effect (Ellis, 2001) in the neuronal cytoplasm. This induces α-synuclein and other naturally unfolded protein aggregation, which may lead to the formation of toxic aggregates, fibrils, and protofibrils.

While synuclein’s role in pathology is thoroughly and systematically investigated, their normal physiological functions still remain debated despite almost 30 years of research. Some progress in unveiling α-synuclein physiological role is achieved due to the discovery of its role in synapsis. Several research teams have demonstrated that α-synuclein is implicated in the modulation of synaptic activity through modulation of vesicle release (Chandra et al., 2005; Burré et al., 2010; Bendor et al., 2013; Zaltieri et al., 2015). Numerous studies demonstrate α-synuclein’s participation in vesicle trafficking, chaperoning of SNARE complex assembly (Bonini and Giasson, 2005; Chandra et al., 2005; Burré et al., 2010; Diao et al., 2013), regulation of synaptic vesicles endocytosis, presynaptic terminal topography (Vargas et al., 2014, 2017), and modulation of dopamine release (Abeliovich et al., 2000). However, the progress in understanding the physiology of β and γ-synucleins is much more limited. β-Synuclein inhibits α-synuclein aggregation caused by several types of lipids as well as α-synuclein nucleation (Brown et al., 2016). γ-Synuclein possesses chaperon activity, is involved in the regulation of monoamine homeostasis and cytoskeleton structure rearrangement. Moreover, it may have similar functions as α–synuclein in the process of synaptic vesicle turnover, neurotransmitter release (Ninkina et al., 2012; Kokhan et al., 2013), and regulation of signaling pathways (Surguchov et al., 1999, 2001, 2017; Surgucheva et al., 2006, 2008; Surguchov, 2008, 2015).

Despite the information presented above, many reviews consider the normal functions of α-synuclein to be unclear and vague, creating an impression that it does not have its own function in living cells, and settles for a supporting role in several pathways. In addition to synaptic functions, α-synuclein participation in several cellular processes has been described, e.g., suppression of apoptosis, regulation of glucose level, modulation of calmodulin level, maintenance of PUFA level, antioxidation, neuronal differentiation, and regulation of dopamine biosynthesis (Emamzadeh, 2016). However, it appears that in all these processes, synucleins are not the major players, but just “ramblers in a crowd” with limited authority and jurisdictions. Still the emerging evidence suggests, that synucleins may have a very important non-canonical function unrelated to their role in synapsis. The nuclear localization of α-synuclein and several new findings point to its essential role in the nucleus related to the regulation of gene expression.

## Synucleins As Modulators of Specific Gene Expression

Gradually new results accumulate, and point out that our knowledge about synuclein’s functions is incomplete, their “social network” is larger than it is currently assumed, and they play a more essential dynamic role in the regulation of intracellular processes. The current understanding of their role in cellular processes is slowly shifting from “ramblers in the crowd” to “cops regulating traffic.” This regulatory role is performed through their interactions with key players governing gene expression, i.e., nucleic acids, transcription and translation factors, and histones. Moreover, synucleins may play a role in epigenetic regulation of cellular processes. Epigenetics applies to external modifications to DNA that alter expression of specific genes without changes in the DNA sequence. It can involve several mechanisms, e.g., DNA methylation, histone modifications, and RNA-associated silencing.

While the researchers still keep working out the details of synuclein’s functions in synapsis and a role in vesicles formation, another page of their story emerges, attracting increasing attention. Several new methods demonstrate that synucleins are “more social” that it has been assumed earlier, and find novel partners in synuclein’s “social network” not only among proteins, but also nucleic acids. Below we present several examples of synuclein’s regulatory role in gene expression.

The first hint suggesting that synucleins regulate gene expression came from the analysis of the effect of their upregulation in cell cultures (Seo et al., 2002; Baptista et al., 2003). Although the effect of elevated synucleins level on expression of specific genes was significant, the exact molecular mechanisms was often unknown. In several studies a direct effect of synucleins on transcription in the nucleus is demonstrated, other publications suggest hypothetic mechanisms explaining this effect. For example, α-synuclein downregulates c-Jun N-terminal kinase protecting cells against oxidative stress, upregulates caveolin-1 expression, and downregulates ERK, affecting the pathogenesis of PD (Hashimoto et al., 2003). In another publication α-synuclein’s effect in regulating apoptosis is reported. It reduced Bcl-xL expression and increased BAX expression (Seo et al., 2002).

In several studies a substitution of one amino acid in α-synuclein dramatically changed its regulatory properties suggesting that this effect was mediated by conformational alterations. For example, α-synuclein altered expression of several families of genes including genes responsible for apoptosis, stress response, transcription regulation, and membrane proteins (Baptista et al., 2003). Significant changes in expression levels are also found for genes responsible for the regulation of dopamine synthesis. Reduced expression of the orphan nuclear receptor Nurr1 suggests that the synergetic regulation of dopamine synthesis occurs through this transcription factor. These alterations in mRNAs levels identified by microarray experiments occurred on transcriptional level and were validated by quantitative RT-PCR. Interestingly, the expression levels of four genes were regulated in opposite directions in cells overexpressing wild-type protein or α-synuclein with A53T substitution (Baptista et al., 2003).

Overexpression of wild-type or mutant α-synuclein interferes with DBH transcription regulation by CRE element in catecholaminergic neurons (Kim et al., 2014). In the nucleus α-synuclein interacts with the DBH promoter including the CRE element, which interferes with forskolin-induced transcription factor CREB binding to the CRE region. Thus, α-synuclein attenuates CRE-mediated transcription of DBH. This interaction is physiologically important, because CREB plays an essential role in survival of neurons by controlling the transcription of several genes implicated in cell protection, such as BDNF (Chalovich et al., 2006). Remarkably, mutant α-synuclein demonstrates higher tendency to nuclear translocation and interaction with the DBH promoter than the wild type.

Another example confirming that the effect of α-synuclein on transcription of specific gene may differ as a result of a single amino acid substitution is recently described (Segura-Ulate et al., 2017). Oligodendroglial cells OLN-93 stably expressing either a human wild type or a mutant A53E α-synuclein (multiple system atrophy associated mutant) reduced BDNF mRNA to practically unmeasurable levels. At the same time, another MSA-associated α-synuclein mutant, caused only a small reduction in BDNF mRNA. Therefore, point mutations in α-synuclein may change not only its physico-chemical properties and propensity to aggregation, but also alter its regulatory function (Segura-Ulate et al., 2017). Moreover, in dopaminergic neurons α-synuclein also reduces the expression of PKCδ to inhibit apoptosis, by decreasing enzymatic activity of p300 HAT.

This effect results in neuroprotection in a α-synuclein expressing dopaminergic cell model as a result of exposure to the Parkinsonian neurotoxicant MPP+. This mechanism involves modulation of both NFκB and p300 signaling pathways in transgenic mice and in neuronal culture (Jin et al., 2011). The effect of α-synuclein on histone acetylation is further described in Chapter 6.

α-Synuclein is colocalized with transcription factor Elk-1 and interacts with MAP kinase pathway (Iwata et al., 2001a,b). These findings point to the role of α-synuclein in the modulation of transcription and signaling pathways.

Another member of the synuclein family, γ-synuclein also interacts with Elk-1 (Surguchov et al., 1999). Furthermore, it plays a role as a modulator of matrix metalloproteinases (MMP-9 and MMP-2) expression upregulating both MMP-9 protein level and activity (3.2 to 7.1-fold, respectively). This upregulation takes place on transcriptional level via the activation of the AP-1 *cis*-elements in MMP-9 promoter (Surgucheva et al., 2003). Further studies demonstrated direct binding of γ-synuclein to transcription factors JunB, MECP2, CREB1, PPAR-gamma, TCEA1, and ATF3 (Surgucheva and Surguchov, 2008). Recent study using yeast two-hybrid screening, identified another transcriptional regulator – polyC binding protein 1 (PCBP1) as a γ-synuclein interacting protein (Hunkele et al., 2016).

## Synuclein – DNA Interactions

### α-Synuclein Binding to DNA

The existence of α-synuclein in the nucleus first found by Maroteaux et al. (1988) and later confirmed by many other studies, encouraged researchers to examine synuclein–DNA interaction and to investigate its possible functions. Several research groups demonstrated that α-synuclein could bind directly to a single copy DNA; this binding is especially efficient if DNA is active in the process of transcription and is not bound to histones (Hegde and Jagannatha Rao, 2003; Hegde et al., 2010; Vasudevaraju et al., 2012). This binding occurs preferentially to GC-box-like sequences (Vasudevaraju et al., 2012; Ma et al., 2014) and may alter properties of both protein and DNA. In particular, α-synuclein induces DNA damage by changing its stability, conformation, and by causing DNA nicking (Padmaraju et al., 2011). In turn, DNA can itself modulate α-synuclein folding (Hegde and Rao, 2007). After binding with double- or single-stranded DNA α-synuclein acquires a highly structured conformation. Circular dichroism studies show that the α-helical content of α-synuclein increases from 5 to 64% upon binding to DNA, whereas the random coil decreases from 95 to 33% (Hegde and Rao, 2007).

Interaction of α-synuclein with linear or supercoiled double-stranded DNA (dsDNA) protects DNA from digestion by restriction endonucleases. Complexes between α-synuclein and DNA as well as assembly of wild-type α-synuclein into fibrils in the presence of linear DNA are revealed by electron microscopy (Cherny et al., 2004).

The presence of synucleins in the nucleus and their binding to DNA provides a possibility that such interaction affects transcription regulation and may change neuronal function (Ma et al., 2014; Surguchov, 2014). In several studies such functional consequences of synuclein – DNA interaction have been demonstrated. For example, in neuroglioma, α-synuclein binds to DNA, and regulates the transcription of genes controlling ubiquitination and other biochemical processes linked to PD (Martins et al., 2011).

### DNA Binding Is a Common Feature of Several Amyloidogenic Proteins

α-Synuclein is not a unique amyloidogenic protein possessing DNA-binding ability. Amyloid-beta (Aβ) peptides and prion proteins also have high DNA binding capacity, suggesting that DNA binding may be a common property of amyloidogenic proteins (Hegde et al., 2010). Binding of α-synuclein and other amyloidogenic proteins to DNA may affect normal DNA functions and cause genetic stress altering the normal pattern of gene expression (Jiménez, 2010). Results supporting this unifying hypothesis were obtained from the investigation which identified DNA aptamers that specifically bind to α-synuclein (Tsukakoshi et al., 2010, 2012). In this study eight aptamers specific for α-synuclein monomers, oligomers, and fibrils were characterized by a competitive screening method. Their nucleotide sequences are not conserved, but all possess guanine-rich sequences which form scaffolds-like G-quadruplex structures. Importantly, some of the aptamers recognizing α-synuclein, are also specific for Aβ oligomers. Dissociation constant for such aptamers binding is in nano- to picomolar range. The structure of these aptamers may give a clue for the search of new medications and biomarkers specific for neurodegenerative diseases. Notably, the aptamer could recognize not only the primary structure of α-synuclein, but also its conformation (Tsukakoshi et al., 2012).

Several studies demonstrate that α-synuclein is able to bind directly to promoter region of specific genes and affect their transcription. For example, α-synuclein binding to a promoter of the mitochondrial transcriptional co-activator PGC-1α, which reduces its expression in response to oxidative stress is described (Siddiqui et al., 2012).

Another member of the synuclein family, γ-synuclein also modulates genes expression by binding to the promoter region of specific genes. For instance, upregulation of matrix metalloproteinases-9 (MMP-9) expression and activity is mediated by γ-synuclein binding to AP-1 sites at the promoter region of the MMP-9 gene (Surgucheva et al., 2003). Under stress conditions a translocation of γ-synuclein to the nucleus decreases outgrowth of neurites more efficiently than α-synuclein overexpression. Thus, γ-synuclein may alter its intracellular localization in response to stress and make appropriate alterations in the gene expression pattern (Surgucheva et al., 2003, 2006).

## Binding of Synuclein to RNA

α-Synuclein binds its own mRNA and prevents initiation of translation (Zanzoni et al., 2013). Interaction of proteins with cognate transcript is a known regulatory mechanism modulating gene expression at the translational level. Usually such mRNA contains a riboswitch – a regulatory segment that interacts with a small molecule, affecting the translation of the proteins encoded by the mRNA (Tucker and Breaker, 2005). However, no riboswitches have been described for synucleins yet. Such autoregulatory control is important for assurance of optimal protein expression levels, and abolition of this normal feedback may lead to various negative consequences (Tai et al., 2004; Hasnat et al., 2007). Autogenous interactions alter gene expression at the translational level and when protein production is elevated, binding to mRNA has a significant inhibitory effect on translation efficiency.

To calculate the propensity of proteins to bind RNA, catRAPID approach was put forward which allows to predict the incidence of autogenous associations in the human proteome (Zanzoni et al., 2013). This method demonstrates that α-synuclein easily binds with cognate mRNA, inhibiting its translation, preventing overexpression and thus supporting the optimal level of protein expression. Since aggregation is intrinsically concentration dependent, it is likely that autogenous interactions play a crucial role in controlling protein homeostasis.

Several recently developed methods are useful to investigate the role of RNA-protein interactions in the pathogenesis of human diseases. CatRAPID is a theoretical framework, which predicts the binding ability of protein and RNA molecules based on physico-chemical properties of nucleotide and amino acid chains. This includes hydrogen bonding, secondary structure, and van der Waals’ forces to predict protein–RNA associations with a high confidence (Bellucci et al., 2011; Cirillo et al., 2013). This approach presents a novel tool in the development of RNA aptamers, a useful therapeutic instrument for the diagnostics and management of neurodegenerative diseases (Lee et al., 2006).

Garcia-Esparcia et al. (2015) described significant alterations in the machinery of protein synthesis at the specific regions of the brains of PD patients which are region- and stage-dependent. These alterations include 18S and 28S rRNA, expression of several mRNAs encoding ribosomal proteins, and altered level of translation factors eIF3 and eEF2. These alterations occur in substantia nigra and in the cerebral cortex and may be linked to a significant elevation of α-synuclein oligomers. However, there is no direct evidence to date proving this association.

Thus, there is growing understanding that interaction of α-synuclein with RNA and other protein-RNA interactions are involved in PD and other neurodegenerative diseases (Anthony and Gallo, 2010; Cirillo et al., 2013).

## Data From Yeast Help to Expand α-Synuclein’s Human Interactome Demonstrating Its Critical Role in the Regulation of Translation

New methodologies are required to reveal how synucleins orchestrate the expression of other genes, to identify new binding partners, and to examine mechanisms of synuclein’s toxicity. Classical methods, such as knockdown of a single member or all three members of the synuclein family, give only limited information about synuclein’s function, confirming their role in synapse structure and physiology. Synuclein’s deficiency causes age-dependent neuronal dysfunction, impaired survival and extensive alterations in synaptic dopamine neurotransmission in the nigrostriatal system (Greten-Harrison et al., 2010; Anwar et al., 2011). The results of these experiments also confirmed the existence of overlapping functions in synuclein family members (Anwar et al., 2011). These findings were important for understanding of synuclein pathology in PD and aging, but synuclein biology and their normal physiological functions remained poorly understood.

An important clue for better understanding of a protein’s physiological function is the elucidation of its physical interactions. To recognize the normal biological functions and role of α-synuclein in neuropathology, several transgenic and viral overexpression models were developed in various organisms, including roundworms (nematode), fruit flies, rodents, and non-human primates. Amazingly, significant progress in the identification of new synuclein binding partners was made in budding yeast *Saccharomyces cerevisiae*, the organism without CNS or brain. Moreover, it does not even have α-synuclein ortholog. Yeast *Saccharomyces cerevisiae* have repeatedly been shown to be suitable for studies of high eukaryotic cell biology, for example, programmed cell death, mitochondria biology (Ter-Avanesyan et al., 1982; Grandin and Charbonneau, 1999; Knott et al., 2008), vesicular trafficking (Littleton and Bellen, 1995), secretory pathway (Malhotra and Emr, 2002), as a model to study neuroprotection at the cellular level (Scheper and Hoozemans, 2009). The method used in the laboratory of Susan Lindquist (Khurana and Lindquist, 2010) and other labs (Braun et al., 2010; Tenreiro et al., 2017) also helped to get better understanding of the mechanisms of α-synuclein toxicity. The ‘humanized’ yeast models for synucleinopathies recapitulate the fundamental properties of the pathology on molecular and cellular level, identified in human diseases. This provides the rationale for engaging the powerful analytical instrument for various high throughput screening approaches in yeast. In yeast, genome-wide analyses can be performed, which enables the un-biased identification of genes and pathways critically modulating (either executing or preventing) the toxicity of α-synuclein (Lan et al., 2011; Tardiff et al., 2014; Khurana et al., 2017). In addition, comprehensive transcriptomic data may be collected from yeast cells expressing various levels of α-synuclein. Usually genetic screens identify response regulators, whereas transcriptomic profiling assays reveal components of metabolic processes. The ResponseNet computational method combines data from the genetic screens and gene expression analyses with the comprehensive knowledge on protein (and gene) interaction information in public yeast data bases (Yeger-Lotem et al., 2009). This generated a novel level of information on the functional α-synuclein gene/protein interaction network. The ResponseNet method allowed to reveal cellular pathways that responded to the toxicity of α-synuclein mapping both previously identified, and unrecognized pathways responding to α-synuclein toxicity. In the response to α-synuclein toxicity four *de novo* predictions identified by ResponseNet were validated, i.e., the nitrosative stress, the TOR pathway, the disruption of the sterol biosynthesis pathway and the mode-of-action of the genetic suppressor Gip2 (Yeger-Lotem et al., 2009).

Recently a new important method, TransposeNet, was developed allowing to combine genome-wide genetic screens in yeast with the comprehensive protein and gene interaction information available in yeast databases, using an improved computational method (Khurana et al., 2017). The method allows identification of protein–protein and protein–DNA interactions using a yeast interactome data. With this approach a functional protein/gene interaction network for α-synuclein has been described. An important step in this method is a transposition of α-synuclein interaction network into a human α-synuclein interaction network. This transposition is based on a significantly improved identification of human homologs of yeast genes, and on the information of the protein and gene interaction networks known from both the human system and from yeast. A very important result brought by the TransposeNet is that the mRNA translation subnetwork is relevant for α-synuclein toxicity in patient-derived neurons.

**FIGURE 1:**
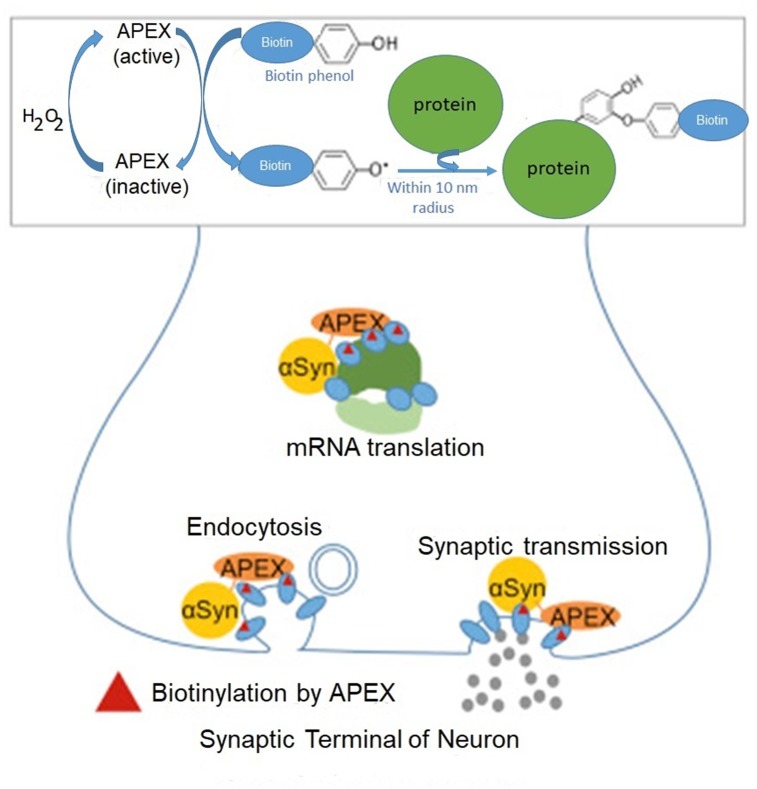
Identification of α-synuclein-interacting proteins by peroxidase labeling technique coupled with mass spectrometry. A genetically engineered peroxidase APEX tagged to the C-terminal of wild-type α-synuclein defines the protein network in the vicinity (10 nm) of α-synuclein in living neurons. In addition to anticipated α-synuclein-interacting proteins involved in synaptic transmission and endocytosis, this method reveals several mRNA binding proteins implicated in mRNA translation. Adapted from Chung et al. (2017) with permission from the copyright holder.

The pivotal role of mRNA translation for α-synuclein toxicity was further substantiated by a biochemical method enriching our knowledge about α-synuclein interactome (Chung et al., 2017). Here, the authors used APEX (from ascorbate peroxidase), a method based on the extremely short-lived radicals which covalently react and label amino acids in their immediate proximity (Martell et al., 2012). This method identifies even transient protein–protein interactions in living neurons by mass spectrometry. The method relies on fusing α-synuclein with APEX, which oxidizes phenol derivatives to phenol radicals (**Figure 1**).

**FIGURE 2:**
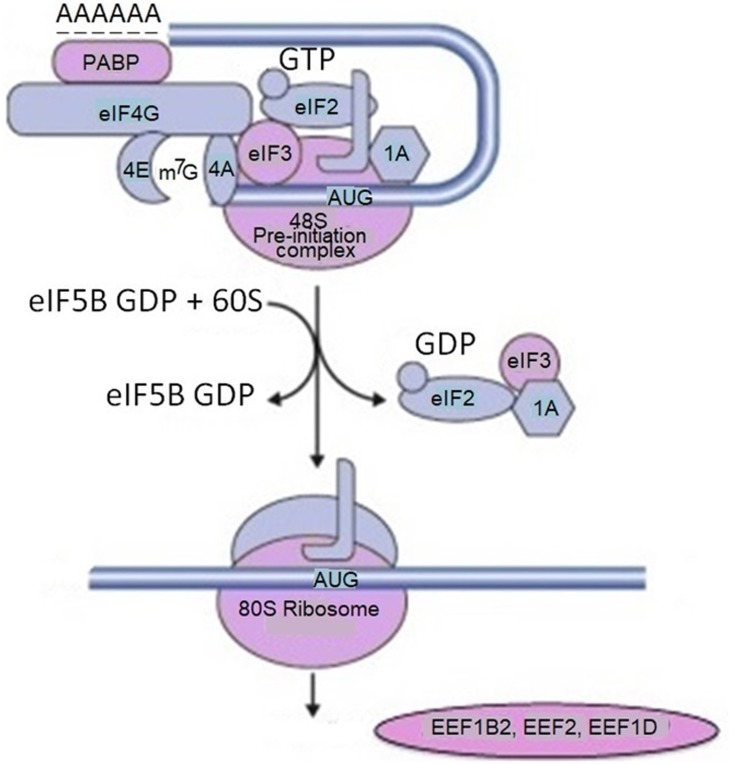
Proteins involved in mRNA translation labeled by α-synuclein-APEX. mRNA translation-related proteins are tested for physical interaction with α-synuclein using the membrane-2-hybrid (MYTH) technique. For example, multiple subunits of translation initiation factor EIF3 are labeled with α-synuclein-APEX and stain positive for MYTH. From the list of mRNA translation hits in the APEX neuron study, five were available for testing by MYTH. Four of those, namely EIF3C, EIF3L, RPS12, and PABPC1 were confirmed. These data imply that mRNA translation machinery elements and binding proteins are in close proximal interactions with α-synuclein. Adapted from Chung et al. (2017) with permission from the copyright holder.

Rat primary cortical neurons were transduced with α-synuclein fused to APEX2, a catalytically superior version of APEX. As a result, α-synuclein interacting proteins were identified by mass spectrometry. In initial screening the authors detected 225 proteins in living neurons which interacted with α-synuclein. Many of these interacting proteins were present in complexes with α-synuclein. This study in addition to anticipated interacting proteins involved in synaptic transmission and endocytosis, identifies several proteins implicated in mRNA metabolism (RNA binding, processing and translation factors). Among them are ten proteins with various functions in translation: EIF3C, EIF3D, CARS, EEF1B2, DARS, EIF3L, EEF2, RPS10, EEF1D and MARS (**Figure 2**).

Polyadenylate-binding protein 1 (PABPC1) – an mRNA binding protein which facilitates mRNA transport out of the nucleus, degradation, translation, and stability is also identified as α-synuclein interacting protein. The authors assume that α-synuclein physically associates with translation factors and sequesters them. This could occur in the synapse, where α-synuclein is localized, and the local mRNA translation plays a key role in the synaptic plasticity. This approach links many Parkinsonism and neurodegenerative disease risk factors to α-synuclein toxicity through specific molecular pathways. The most important of them are mRNA metabolism, translation, and vesicle trafficking. In this network, α-synuclein is associated with genetic modifiers related to mRNA translation, including initiation factors. In particular, it binds to translation initiation factor 4 gamma 1 (EIF4G1) and the poly(A)-binding protein (PAB or PABPC1). The latter one binds to the 3′ poly(A) tail of mRNA and is involved in poly(A) shortening and translation initiation. In this network α-synuclein is also associated with several ribosomal components. Overexpression of these genetic modifiers in the mRNA translation and mRNA processing pathways suppressed α-synuclein toxicity in bioscreen, while genetic experiments in various disease models revealed that their effects were specific. As a result, α-synuclein screens and network analysis identified a robust effect of α-synuclein toxicity on bulk mRNA translation in cellular models of synucleinopathy, which was not attributable to an ER stress response (Khurana et al., 2017). The link of α-synuclein with EIF4G1 presumably is involved in pathological alterations in PD and other neurodegenerative disease, since this translation initiation factor is linked to both PD (Chartier-Harlin et al., 2011) and Lewy body dementia (Fujioka et al., 2013). EIF4G1 functions as a scaffold in the eIF4F initiation complex, recruiting other components of translation machinery, i.e., ribosomes and tRNAs to the 5′ cap of mRNA (Sonenberg and Dever, 2003).

## α-Synuclein Regulates Cellular Processes by Epigenetic Mechanisms

### Interaction of α-Synuclein with Histones

The first evidence of α-synuclein interaction with histones was published almost 15 years ago (Goers et al., 2003). The initial data describing this interaction were obtained from the studies of neurotoxicity. After injections of the herbicide paraquat to mice α-synuclein was colocalized with histones in the nuclei of nigral neurons (Goers et al., 2003; Cherny et al., 2004). α-Synuclein formed a tight complex with histones with a molecular mass of 48,700 Da, with a stoichiometry of 2:1 (α-synuclein/histone), and a dissociation constant of about 1 μM. Hypothetically, histones enriched with arginine and lysine residues act as scaffolds bringing together molecules of α-synuclein with high content of acidic amino acids at C-terminal domain. The authors assume that translocation into the nucleus and binding of histones is one of the mechanisms underlying α-synuclein toxicity (Goers et al., 2003).

### Physiological Consequences of α-Synuclein-Histone Interaction

Further studies demonstrate that interaction of α-synuclein with histones has an important physiological function. Binding of α-synuclein to histones decreases the histone H3 acetylation and reduces acetylation in HAT assays (Kontopoulos et al., 2006). Two α-synuclein mutations, A30P and A53T, that cause familial PD, display increased probability of localization in the nucleus. These data point to an importance of further research on histone deacetylase inhibitors as a potential target for the treatment of this disease.

Several lines of evidence show that interaction of α-synuclein with histones may also alter transcription of specific genes, representing epigenetic mechanism of gene expression regulation. α-Synuclein overexpression enhances mono- and dimethylation of histone H3K9, resulting in an increase in methylated form of this histone at the SNAP25 promoter, presumably disturbing SNARE complex assembly and fusion of synaptic vesicles (Sugeno et al., 2016). Histone modification is an epigenetic mechanism which has a unique role in the cell, modulating the transactivation or repression of particular genes. Such mechanism may contribute to synaptic dysfunction occurring in PD (Sugeno et al., 2016).

Another group examined the role of histones in α-synuclein aggregation in cells experiencing apoptosis and in neuronal cells with nuclear membrane defects causing leakiness. The authors found that histones H1 and H3 released into the cytoplasm during apoptosis interact with α-synuclein to form cytoplasmic aggregates and play a role as a proaggregant factor. Importantly, their results demonstrate that histone-induced α-synuclein aggregates are transmissible to neurons both *in vitro* and *in vivo*. Histone-induced α-synuclein aggregates could spread to neurons and seed α-synuclein aggregation. Histone H1 less tightly associated with DNA is more important in the formation of pathological forms of α-synuclein. Histone-induced aggregates contain α-synuclein oligomers of variable size, including protofibrils and mature fibrils. The authors hypothesize that endogenous histones might facilitate internalization of α-synuclein aggregates and favor cell-to-cell propagation similar to the widely used transfection reagent lipofectamine (Jiang et al., 2017).

**FIGURE 3:**
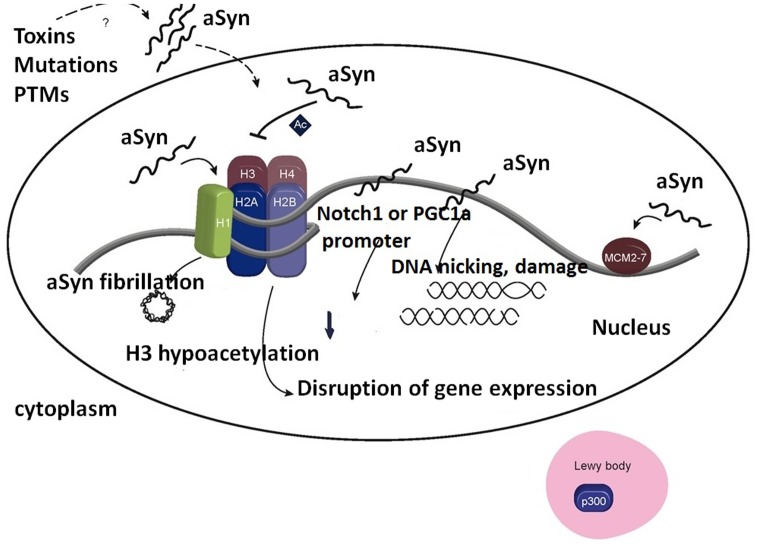
Association of α-synuclein with histones in the cell nucleus induces formation of α-synuclein fibrils. Importantly, inhibition of H3 histone acetylation by α-synuclein affects the expression level of genes responsible for cell survival. Furthermore, association of α-synuclein with p300 decreases HAT activity. Binding of α-synuclein to DNA affects the expression of several genes, e.g., NOTCH1 and PGC-1A, and may lead to DNA damage. It allows the preservation of associated MCM2-7 complexes on the chromatin. Ac, acetylation; PTMs, post-translational modifications. Adapted from Pavlou et al. (2017) with permission from the copyright holder.

Importantly, binding of α-synuclein to histones not only affects histone modifications (**Figure 3**), but also it accelerates α-synuclein fibrillation.

Synuclein binding with histone H1 or the other core histones, causes the enhancement of α-synuclein fibrillation. α-Synuclein is able to reduce H3 acetylation, affecting the expression of several genes responsible for cell survival (Pavlou et al., 2017).

### Effect of α-Synuclein on DNA Methylation

Another mechanism of α-synuclein participation in epigenetic regulation is realized via DNA methylation. α-Synuclein associates with Dnmt1 causing mislocalization and retention of Dnmt1 in the cytoplasm of neuronal cells (Desplats et al., 2011). As a result methylation in the regulatory regions of specific genes in PD and DLB brains is significantly reduced. The effect is observed both in post-mortem human brain samples and in brains of animal models of PD/DLB. Importantly, overexpression of another member of the synuclein family, β-synuclein does not cause cytoplasmic retention and Dnmt1 is not immunoprecipitated by anti-β-synuclein antibody, confirming the specificity of association between α-synuclein with Dnmt1. However, this effect could be reversed by lentivirus-mediated overexpression of Dnmt1. The finding of the inverse relation between α-synuclein oligomerization and Dnmt1 content in the nucleus suggests that the sequestration of Dnmt1 is increased by α-synuclein aggregation (Desplats et al., 2011). The loss of Dnmt1 protein from the cell nuclei is described in brains of patients with PD, DLB, and brains from transgenic mice that overexpress α-synuclein. In turn, these changes lead to the alterations in methylation pattern at the promoters of α-synuclein and several other genes, associated with neurodegenerative diseases (Desplats et al., 2011). A reduction in Dnmt1 causing mislocalization has been described in PD patient’s brains. Another team (Jowaed et al., 2010) found that methylation in intron 1 decreases α-synuclein expression, whereas inhibition of DNA methylation upregulates expression. Furthermore, methylation of intron 1 in α-synuclein gene is reduced in DNA from sporadic PD patients’ brain, including substantia nigra, putamen, and cortex. These results confirm epigenetic regulation of α-synuclein expression in PD (Jowaed et al., 2010). This data suggest another possible mechanism of α-synuclein interference with pathogenic processes of PD.

### Epigenetic Mechanisms Regulating α-Synuclein Expression May Affect Its Regulatory Functions

As discussed in chapter 2, α-synuclein role in gene expression regulation depends on its intracellular concentration which in turn is regulated by epigenetic mechanisms controlling its levels. These mechanisms include methylation of α-synuclein promoter, post-translational modifications of histones, and epigenetic mechanisms based on non-coding RNAs (Pavlou et al., 2017).

Epigenetic mechanisms that control α-synuclein expression level may affect both its aggregation state and regulatory properties. Importantly, methylation level of CpG sites in α-synuclein gene in leukocytes correlates with the level in brain cells and therefore this analysis may be used as informative biomarker for prognosis of neurodegenerative diseases (Tan et al., 2014; Schmitt et al., 2015).

### α-Synuclein and Chromatin Remodeling

The molecular interactions described above in Chapter 6, including binding of α-synuclein with histones, effect on DNA methylation, and epigenetic mechanisms could all induce chromatin remodeling.

Association of α-synuclein with histones may disturb nucleosome structure by reducing the availability of free histones for binding with DNA, or by affecting histone PTMs and leading to alterations in the pattern of gene transcription. In post-mortem PD brains the association of α-synuclein and chromatin is higher than in control samples. Furthermore, α-synuclein binds to the promoter of the master mitochondrial transcription activator, PGC1 alpha (PGC-1α), which is downregulated in PD brains (Siddiqui et al., 2012; Pavlou et al., 2017). The role of α-synuclein in chromatin remodeling is discussed in a recent review (Pavlou et al., 2017).

## Conclusion

Here we discuss recent findings showing that in addition to their role in synapses, synucleins have non-canonical functions, and are involved in the regulation of essential cellular processes. In particular, the results presented here are consistent with a significant, although not completely understood role of synucleins in the regulation of gene expression. These mechanisms include both direct interaction of synucleins with DNA, transcription and translation factors, and less direct intervention, for example, via their effect on histone acetylation. Thus, synucleins may play a passive role of “ramblers in the crowd,” but in response to stress or changing environmental conditions they become involved in the modulation of specific protein expression via mechanisms described in Chapters 2–4 and 6. Further research is required to unveil the details of these intimate mechanisms.

## Author Contributions

AAS wrote, and edited the manuscript; made substantial contributions to the conception of the work; made a final approval; and agreed to be accountable for all aspects of the work. AS wrote a part of the manuscript; made analysis and interpretation of data; made a final approval of the version to be published; and agreed to be accountable for all aspects of the work.

## Conflict of Interest Statement

The authors declare that the research was conducted in the absence of any commercial or financial relationships that could be construed as a potential conflict of interest.

## Abbreviations

Aβ: amyloid beta
BDNF: brain-derived neurotrophic factor
DBH: dopamine-β-hydroxylase
DLB: dementia with Lewy Bodies
DNMT: DNA methyltransferase
eEF2: translation elongation factor 2
eIF3: translation initiation factor 3
HAT: histone acetyltransferase
MMP: matrix metalloproteinase
PD: Parkinson’s disease
PKCδ: protein kinase Cδ
